# DPP-4 inhibition with linagliptin ameliorates the progression of premature aging in klotho−/− mice

**DOI:** 10.1186/s12933-017-0639-y

**Published:** 2017-12-01

**Authors:** Yu Hasegawa, Kenyu Hayashi, Yushin Takemoto, Cao Cheng, Koki Takane, Bowen Lin, Yoshihiro Komohara, Shokei Kim-Mitsuyama

**Affiliations:** 1 Department of Pharmacology and Molecular Therapeutics, Graduate School of Medical Sciences, Kumamoto University, 1-1-1, Honjo, Chuo-ku, Kumamoto-Shi, Kumamoto-ken 8608556 Japan; 2 Department of Cell Pathology, Graduate School of Medical Sciences, Kumamoto University, Kumamoto, Japan

**Keywords:** Premature aging, DPP-4, Klotho−/− mice, Cognition, Brain aging, Pleiotropic effect

## Abstract

**Background:**

The potential of anti-aging effect of DPP-4 inhibitors is unknown. This study was performed to determine whether linagliptin, a DPP-4 inhibitor, could protect against premature aging in klotho−/− mice.

**Methods:**

Klotho−/− mice exhibit multiple phenotypes resembling human premature aging, including extremely shortened life span, cognitive impairment, hippocampal neurodegeneration, hair loss, muscle atrophy, hypoglycemia, etc. To investigate the effect of linagliptin on these aging-related phenotypes, male klotho−/− mice were divided into two groups: (1) control group fed the standard diet, and (2) linagliptin group fed the standard diet containing linagliptin. Treatment with linagliptin was performed for 4 weeks. The effect of linagliptin on the above mentioned aging-related phenotypes was examined.

**Results:**

Body weight of klotho−/− mice was greater in linagliptin group than in control group (11.1 ± 0.3 vs 9.9 ± 0.3 g; P < 0.01), which was associated with greater gastrocnemius muscle weight (P < 0.01) and greater kidney weight (P < 0.05) in linagliptin group. Thus, linagliptin significantly prevented body weight loss in klotho−/− mice. Survival rate of klotho−/− mice was greater in linagliptin group (93%) compared to control group (67%), although the difference did not reach statistical significance (P = 0.08). None of linagliptin-treated klotho−/− mice had alopecia during the treatment (P < 0.05 vs control klotho−/− mice). Latency of klotho−/− mice in passive avoidance test was larger in linagliptin group than in control group (P < 0.05), indicating the amelioration of cognitive impairment by linagliptin. Cerebral blood flow of klotho−/− mice was larger in linagliptin group than in control group (P < 0.01), being associated with greater cerebral phospho-eNOS levels (P < 0.05) in linagliptin group. Neuronal cell number in hippocampal CA1 region was greater in linagliptin group than in control group (P < 0.05). Linagliptin group had greater cerebral phospho-Akt (P < 0.05) and phospho-CREB (P < 0.05) than control group. Thus, linagliptin ameliorated brain aging in klotho−/− mice. The degree of hypoglycemia in klotho−/− mice was less in linagliptin group than in control group, as estimated by the findings of OGTT.

**Conclusions:**

Out work provided the evidence that DPP-4 inhibition with linagliptin slowed the progression of premature aging in klotho−/− mice, and provided a novel insight into the potential role of DPP-4 in the mechanism of premature aging.

## Introduction

Dipeptidyl peptidase-4 (DPP-4) inhibitors are widely used blood glucose-lowering drug for treatment of type 2 diabetes [1, 2]. DPP-4 inhibitors block the degradation of glucagon-like peptide-1 (GLP-1) and gastric inhibitory peptide (GIP), thereby prolonging the half-life of these incretins. DPP-4 inhibitors are modestly effective in reducing HbA1c and have a neutral effect on body weight. Interestingly, accumulating experimental data [2–4] including our reports [5, 6] support that DPP-4 inhibitors have pleiotropic protective effects against cardiovascular and brain injuries independently of blood glucose-lowering effect. Pooled and meta-analyses with individual DPP-4 inhibitors [3, 4, 7] and a pooled analysis of all DPP-4 inhibitors [8, 9] demonstrated significant reduction of cardiovascular disease by DPP-4 inhibitors, although recent two large clinical trials [10, 11] suggested a neutral effect of this class of drug on cardiovascular endpoints. Thus, at present, the benefit of DPP-4 inhibitors in prevention of cardiovascular morbidity and mortality remains to be defined.

Type 2 diabetes is well known to accelerate premature aging in humans as well as the progression of cardiovascular diseases. Therefore, it is a key issue whether DPP-4 inhibitors can exert protective effect against premature aging. In spite of extensive previous animal studies [2–4] indicating the pleiotropic effects of DPP-4 inhibitors, the potential of anti-aging effects of DPP-4 inhibitors is unknown. The klotho gene was identified in 1997 [12]. Klotho−/− mice exhibit multiple phenotypes resembling human premature aging, including extremely shortened life span, cognitive impairment, hippocampal neurodegeneration, hair loss, atrophy of skin and muscle, ectopic calcification, osteoporosis, etc. [12–18]. Klotho−/− mice are one of the best characterized and established animal models of human premature aging. However, to our knowledge, there is no report investigating the effect of DPP-4 inhibitor on klotho−/− mice.

In the present study, to address the potential role of DPP-4 in premature aging, we examined the effect of linagliptin on premature aging phenotypes in klotho−/− mice. We provided the evidence that DPP-4 inhibition ameliorated the progression of premature aging in klotho−/− mice.

## Methods

### Experimental animals

All experiments were approved by the institutional Animal Care and Use Committee of Kumamoto University. Male homozygous mutant klotho−/− mice and C57BL6J mice (wild-type mice) were purchased from Nihon CLEA (Tokyo, Japan). In the present study, according to the instruction of the supplier of these mice, 2 klotho−/− mice and 2 wild-type mice were housed in one cage, since klotho−/− mice are extremely vulnerable to stress and individual housing of klotho−/− mice significantly shortens life span.

### Drugs

Linagliptin was kindly provided by Boehringer Ingelheim (Ingelheim, Germany).

### Experimental protocol

**Fig. 1:**
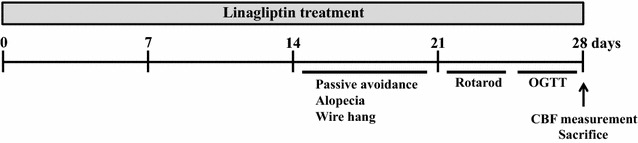
Study protocol. The days when each test was performed are indicated at the line. OGTT, oral glucose tolerance test; CBF, cerebral blood flow

Five-week-old male klotho−/− mice were divided into 2 groups. Group 1 (n = 15) of mice were fed standard diet (MF diet, ORIENRAL YRAST Co., Ltd., Tokyo, Japan) and group 2 (n = 15) of mice were fed the standard diet containing linagliptin (0.083 g/kg diet). This dose of linagliptin is shown to be an appropriate dose for estimation of pharmacological action of linagliptin in vivo, as shown by previous reports [5, 6, 19, 20]. Drug treatment was performed for 4 weeks (from 5 to 9 weeks of age). Survival of each mouse was monitored every day and body weight was monitored every week. Passive avoidance test, monitoring of alopecia, wire-hang test, rotarod test, and oral glucose tolerance test (OGTT) were performed at specified time point as shown in the detailed experimental protocol (Fig. 1). After 4 weeks of the drug treatment, the mice had measured cerebral blood flow (CBF) under 1.5–2% isoflurane, had blood samples taken from the right ventricle, and perfused with phosphate-buffered saline. Subsequently, various organs including cerebrum, cerebellum, left and right ventricle, kidney, subcutaneous and visceral fat, and gastrocnemius muscle were removed and weighed. Then, rostral side from bregma of each cerebrum was immediately frozen. Caudal side from bregma of each cerebrum and kidney was immediately frozen in Tissue-tek OCT embedding medium (Sakura Finetek, Tokyo, Japan). Gastrocnemius muscle was embedded in paraffin. An 8-µm slice was made at 1.43–2.43 mm caudally from the bregma for the following histological evaluations. In addition, 8-µm slice of kidney and 5-µm slice of gastrocnemius muscle were made for the following histological evaluations.

Wild-type mice (C57BL6J) were also divided into 2 groups (n = 10 in each group) and received linagliptin treatment in the same manner as klotho−/− mice.

### Passive avoidance test

The passive avoidance test consisted of two sections including (1) training section and (2) memory test section (Muromachi Kikai, Tokyo, Japan), as previously described by Ma et al. [6]. Briefly, in the training section, the mice were given the electric shock (1.6 mA for 3 s) and kept in the dark side of the test box for 60 s, and this step was repeated three times. In the memory test section, the mice were put into the light side of the test box at 1 day after the training section, and recorded the seconds until the mice went into the dark place (for up to 300 s).

### Alopecia scoring

Alopecia was scored by visual assessment as follows; whole body of the mice was divided into 4 segments (dorsal and ventral portion of the body, head, and extremities) and each part of obvious alopecia was scored 1. The maximum score is 4 and higher scores indicate severe alopecia.

### Wire hang test

Wire hang test was carried out to assess motor strength. The mice were placed on wire mesh and allowed to grasp their four limb hanging. Then, the wire mesh was turned upside-down gently and the latency were recorded until the mice fell off to a ground (for up to 60 s).

### Rotarod test

Rotarod test was done to assess sensorimotor coordination and motor strength as previously described by Hasegawa et al. [21]. Briefly, animals were placed on the rotating spindle (MK-630B, Muromachi Kikai Co., Ltd., Tokyo, Japan) and a training session were performed at a constant race of 4 rotation per minute (RPM) for 1 min. Then, the mice were subjected to the trial on the accelerating spindle (4–40 RPM) and the latency was recorded until the mice fell off from the cylinder. The mean times for three trial of the test were assigned for each animal.

### Oral glucose tolerance test (OGTT) and measurement of non-fasting blood glucose level

The oral glucose tolerance test was performed as previously described [6, 22]. Briefly, the mice were fasted for 6 h and given glucose (1 mg/g body weight) orally. The blood glucose concentration collected from tail vein was measured at 0, 30, 60, and 120 min after glucose administration using portable glucose meter (Sanwa Kagaku Kenkyusho Co., Ltd., Nagoya, Japan).

### Measurement of cerebral blood flow

The baseline CBF of the mice was measured using a laser speckle blood flow imager (Omega Zone; Omegawave, Tokyo, Japan), as described by Toyama et al. [23].

### Measurement of serum DPP-4 activity, insulin, and glucose levels

Serum DPP-4 activity, insulin and glucose concentrations at the end of drug treatment were evaluated. DPP-4 activity was measured using DPP-4-Glo™ Protease Assay (Promega Corporation, Madison, WI, USA) as previously described by Ma et al. [6]. Insulin concentration was measured using a commercial ELISA kit (Morinaga Institute of Biological Science, Inc., Kanagawa, Japan). Non-fasting serum glucose concentration was evaluated using a Glucose C2 test kit (Wako Pure Chemical Industries, Ltd., Osaka, Japan).

### Nissl staining

To assess the number of hippocampal neuronal cells, we stained two slices which were randomly selected from the brain slices and then bilateral hippocampal CA1 neuronal cells at 200× magnification in each slice were counted. The average number of the cells from the 4 regions was compared between the groups.

### Western blot

The rostral side of right hemisphere was used for western blot analysis according to our previous method [24]. The following primary antibodies were used: anti-phosphorylated Akt and anti-Akt (1:2000, Cell Signaling Technology, Danvers, MA, USA), anti-phosphorylated eNOS (1:1000, BD Transduction Laboratories, Tokyo, Japan), anti-eNOS (1:2000, Cell Signaling Technology), anti-phosphorylated cAMP response element binding protein (CREB) (1:1000, Cell Signaling Technology), and anti-CREB (1:300, Cell signaling Technology) antibodies. The intensity of those bands was quantified using Image J software (National Institutes of Health, Bethesda, MD, USA).

### Measurement of renal calcification

To detect renal calcification, the kidney sections were stained using the von Kossa methods according to the manufacturer’s protocol (Polysciences, Warrington, PA, USA). The deposit area of calcification was calculated by WinROOF version 5.8 analysis software (Mitani Corporation, Fukui, Japan) using 4 pictures at 40× magnification in each section.

### Measurement of renal fibrosis

Eight-micrometer slices from kidney were stained with Sirius Red F3BA (0.5% wt/vol in saturated aqueouspicric acid, Aldrich Chemical Company, St. Louis, MO, USA) as previously described by Lin et al. [25]. The area of fibrosis was taken a picture at 200× magnification and analyzed by WinROOF version 5.8 analysis software (Mitani Corporation), and expressed as a percentage per region of interest.

### Measurement of gastrocnemius muscle fiber size

The muscle samples with paraffin were stained with hematoxylin and eosin. To evaluate the muscle fiber size, thirty muscle fibers were measured by Image J software (National Institutes of Health) using the method of minimum Feret diameter and the mean diameter were compared between the groups as previously described by Briguet et al. [26].

### Statistical analysis

All data were expressed as the mean ± SEM. The statistical analyses were evaluated using Graphpad prism version 6 (Graphpad Software, San Diego, CA, USA) and Statcel (OMS Publication, Saitama, Japan). The mortality of the animals was analyzed by a standard Kaplan–Meier analysis with a log rank test and χ^2^ analysis. Evaluations collected over time in OGTT were done using one-way repeated measurement ANOVA with a Bonferroni correction. Parametric and non-parametric evaluations were performed by a non-paired t test or Mann–Whitney U test in two groups. Unpaired t test was used only when a normal distribution is confirmed in two groups and similar variances are obtained between two groups. If two groups do not meet these requirements, Mann–Whitney test was used as an appropriate method. Differences of P < 0.05 were considered significant.

## Results

### Effects of linagliptin on body weight, survival rate, and alopecia of klotho−/− mice

**Fig. 2:**
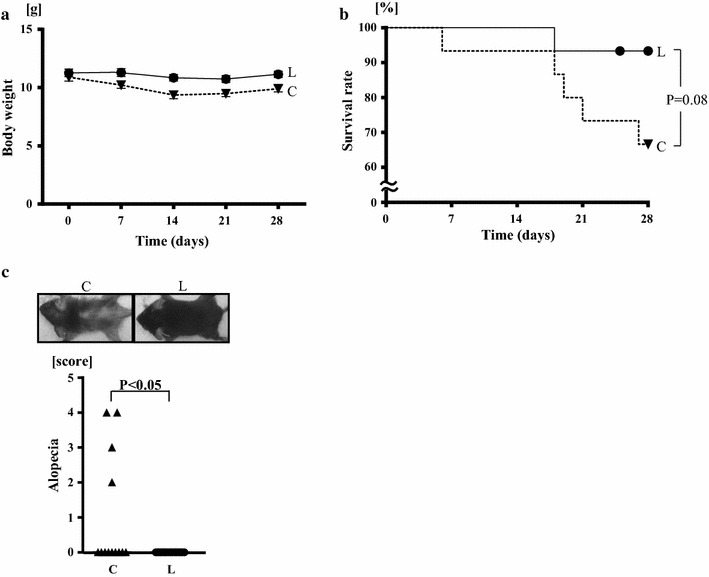
Effects of linagliptin treatment on body weight (**a**), survival rate (**b**), and alopecia (**c**) of klotho−/− mice. C, control group; L, linagliptin-treated group. Upper panels in **c** indicate the appearance of both groups of mice. Values are mean ± SEM. In **a**, **b** n = 15 in each group at day 0. In **c** n = 13 in control group, n = 15 in linagliptin-treated group. In **b** statistical significance was analyzed by a standard Kaplan–Meier analysis with a log rank test and χ^2^ analysis. In **c** statistical analysis was performed by Mann–Whitney U test

As shown in Fig. 2a and Table 1, body weight after 4 weeks of the treatment (at the end of the treatment) was significantly greater in linagliptin group than in control group (11.1 ± 0.3 vs 9.9 ± 0.3 g; P < 0.01). On the other hand, tibia length at the end of the treatment was similar between the groups (15.3 ± 0.1 vs 15.1 ± 0.2 mm), suggesting that linagliptin did not significantly affect the growth of klotho−/− mice (Table 1).

**Table 1:** Effect of 4 weeks of linagliptin treatment on body weight, tibia length, and organ weights in klotho (−/−) mice

	Control (n = 10)	Linagliptin (n = 14)	Statistical significance
Body weight (g)	9.9 ± 0.3	11.1 ± 0.3	P < 0.01
Tibia length (mm)	15.1 ± 0.2	15.3 ± 0.1	NS
Cerebrum/TL (mg/mm)	19.3 ± 0.2	19.0 ± 0.2	NS
Cerebellum/TL (mg/mm)	3.6 ± 0.1	3.6 ± 0.1	NS
Left ventricle/TL (mg/mm)	2.5 ± 0.1	2.6 ± 0.1	NS
Right ventricle/TL (mg/mm)	0.6 ± 0.03	0.7 ± 0.04	NS
Kidney/TL (mg/mm)	4.6 ± 0.2	5.2 ± 0.2	P < 0.05
Subcutaneous fat/TL (mg/mm)	2.8 ± 0.6	3.9 ± 0.5	NS
Visceral fat/TL (mg/mm)	3.1 ± 0.5	4.3 ± 0.7	NS
Gastrocnemius muscle/TL (mg/mm)	2.9 ± 0.1	3.4 ± 0.1	P < 0.01

Values are mean ± SEM. Statistical analysis was performed by non-paired t test or Mann–Whitney U test

Control, control group; Linagliptin, linagliptin-treated group; TL, tibia length; NS, not significant

As shown in Fig. 2b, survival rate of klotho−/− mice at the end of the treatment was greater in linagliptin group (93%) compared to control group (67%), although the difference did not reach statistical significance (P = 0.08).

As shown in Fig. 2c, 4 mice of control klotho−/− mice exhibited alopecia 3 weeks after the start of the treatment, while none of linagliptin-treated klotho−/− mice had alopecia (P < 0.05 vs control klotho−/− mice).

### Effects of linagliptin on cognitive function, cerebral blood flow, hippocampal neuronal cell, and brain Akt, eNOS, and CREB of klotho−/− mice

**Fig. 3:**
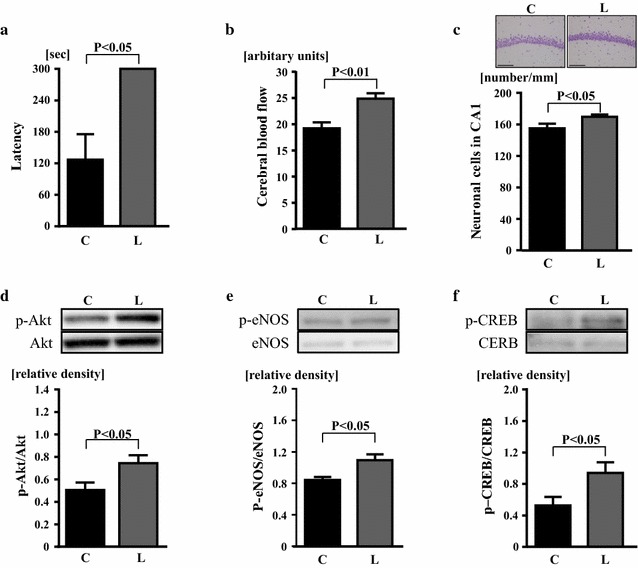
Effects of linagliptin treatment on latency of passive avoidance test (**a**), resting cerebral blood flow (**b**), hippocampal CA1 neuronal cell number (**c**), cerebral phospho-Akt (**d**), cerebral phospho-eNOS (**e**), and cerebral phospho-CREB (**f**) of klotho−/− mice. Abbreviations used are the same as in Fig. 2. Upper panels in **c** indicate representative photomicrographs of Nissl-stained hippocampal sections of each group. Bar = 100 μm. In **d**, **e** upper panels show representative western blot bands. Values are mean ± SEM. In **a**, **c** n = 7 in each group. In **b** n = 10 in control group, n = 12 in linagliptin-treated group. In **d**–**f** n = 6 in each group. In **a** statistical analysis was performed by Mann–Whitney U test. In **b**–**f** statistical analysis was performed by non-paired t test

As shown in Fig. 3a, latency of klotho−/− mice in passive avoidance test was significantly larger in linagliptin group than in control group (P < 0.05). CBF of klotho−/− mice was larger in linagliptin group than in control group (P < 0.01) (Fig. 3b). Neuronal cell number in hippocampal CA1 region was greater in linagliptin group than in control group (P < 0.05) (Fig. 3c). Linagliptin group had greater cerebral phospho-Akt (P < 0.05), greater cerebral phospho-eNOS (P < 0.05), and greater cerebral phospho-CREB (P < 0.05) levels than control group (Fig. 3d–f).

### Effects of linagliptin on serum DPP-4 activity, non-fasting blood glucose and insulin, glucose tolerance of klotho−/− mice

**Fig. 4:**
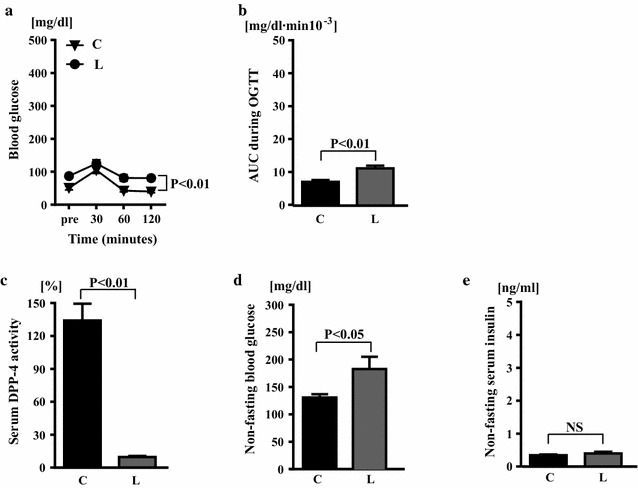
Effects of linagliptin treatment on glucose tolerance (**a**), area under curve (AUC) during oral glucose tolerance test (**b**), serum DPP-4 activity (**c**), non-fasting blood glucose (**d**), and non-fasting serum insulin (**e**) of klotho−/− mice. Abbreviations used are the same as in Fig. 2. NS, not significant. Values are mean ± SEM. In **a**–**c** n = 7 in each group. In **d** n = 8 in control group, n = 10 in linagliptin-treated group. In **e** n = 6 in control group, n = 7 in linagliptin-treated group. In **a** statistical analysis was performed by one-way repeated measurement ANOVA with Bonferroni correction. In **b**, **d**, **e** statistical analysis was performed by non-paired t test. In **c** statistical analysis was performed by Mann–Whitney U test

Blood glucose levels of klotho−/− mice during OGTT were higher in linagliptin group than in control group (P < 0.01) (Fig. 4a), and AUC during OGTT was significantly greater in linagliptin group than in control group (11.1 ± 0.80 vs 7.03 ± 0.46 mg/dl min 10^−3^; P < 0.01) (Fig. 4b).

As shown in Fig. 4c, serum DPP-4 activity of klotho−/− mice was much less in linagliptin group than in control group (P < 0.01). Non-fasting blood glucose levels of klotho−/− mice were greater in linagliptin group than in control group (183.0 ± 22.6 vs 131.3 ± 6.1 mg/dl; P < 0.05), while non-fasting serum insulin levels were not significantly different between the groups (0.40 ± 0.05 vs 0.34 ± 0.28 ng/ml) (Fig. 4d, e).

### Effects of linagliptin on motor activity, skeletal muscle, kidney, and muscle strength of klotho−/− mice

**Fig. 5:**
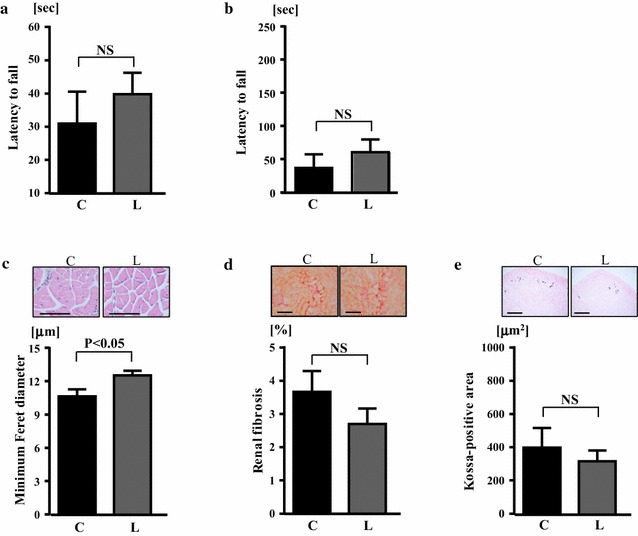
Effects of linagliptin treatment on latency to fall of wire hang test (**a**), latency to fall in rotarod test (**b**), minimum Feret diameter of gastrocnemius muscle (**c**), renal fibrosis (**d**), and renal calcification (**e**) of klotho−/− mice. Abbreviations used are the same as in Fig. 2. NS, not significant. Upper panels in **c** indicate representative photomicrographs (×400 magnification) of hematoxylin and eosin-stained sections of gastrocnemius muscle from both groups. Upper panels in **d**, **e** indicate representative photomicrographs of Sirius red-stained sections and Kossa-stained sections, respectively, of the kidney from both groups of klotho−/− mice. Bar indicates 100 μm in **c**, **d** and 500 μm in **e**. Values are mean ± SEM. n = 7 in each group. In **a**, **b**, **d**, **e** statistical analysis was performed by non-paired t test. In **c** statistical analysis was performed by Mann–Whitney U test

Falling latency in wire hang test and rotarod test in klotho−/− mice were not significantly different between linagliptin and control groups (Fig. 5a, b). However, as shown in Table 1, gastrocnemius muscle weight of klotho−/− mice was greater in linagliptin group than in control group (3.4 ± 0.1 vs 2.9 ± 0.1 mg/mm tibia length; P < 0.01). In addition, minimum ferret diameter of gastrocnemius muscle was also significantly greater in linagliptin group than in control group (12.5 ± 0.42 vs 10.6 ± 0.63 μm; P < 0.05) (Fig. 5c). As shown in Table 1, kidney weight of klotho−/− mice was greater in linagliptin group than in control group (5.2 ± 0.2 vs 4.6 ± 0.2 mg/mm tibia length; P < 0.01). However, the degree of renal fibrosis and renal calcification of klotho−/− mice were not significantly different between control and linagliptin groups (Fig. 5d, e).

### Effects of linagliptin on wild-type mice

As shown in Table 2, treatment of wild-type mice with linagliptin did not significantly affect body weight, tibia length, or organ weights including kidney weight and gastrocnemius muscle weight.

**Table 2:** Effect of 4 weeks of linagliptin treatment on body weight, tibia length, and organ weights in wild-type mice

	Control (n = 10)	Linagliptin (n = 10)	Statistical significance
Body weight (g)	27.2 ± 1.4	27.0 ± 1.7	NS
Tibia length (TL, mm)	17.0 ± 0.1	17.0 ± 0.1	NS
Cerebrum/TL (mg/mm)	20.5 ± 0.2	20.4 ± 0.2	NS
Cerebellum/TL (mg/mm)	3.6 ± 0.1	3.7 ± 0.1	NS
Left ventricle/TL (mg/mm)	5.6 ± 0.2	5.7 ± 0.1	NS
Right ventricle/TL (mg/mm)	1.3 ± 0.1	1.4 ± 0.1	NS
Kidney/TL (mg/mm)	9.7 ± 0.1	9.2 ± 0.5	NS
Subcutaneous fat/TL (mg/mm)	22.0 ± 1.3	18.6 ± 1.5	NS
Visceral fat/TL (mg/mm)	53.8 ± 4.3	58.6 ± 4.5	NS
Gastrocnemius muscle/TL (mg/mm)	8.8 ± 0.3	8.5 ± 0.1	NS

Abbreviations used are the same as in Table 1. Values are mean ± SEM. Statistical analysis was performed by non-paired t test or Mann–Whitney U test

**Fig. 6:**
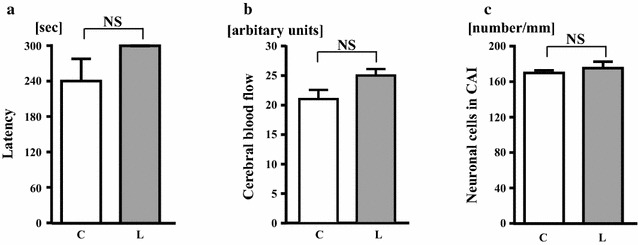
Effects of linagliptin treatment on latency of passive avoidance test (**a**), resting cerebral blood flow (**b**), and hippocampal CA1 neuronal cell number (**c**) of wild-type mice. Abbreviations used are the same as in Fig. 2. Values are mean ± SEM. In **a**, **c** n = 7 in each group. In **b** n = 10 in each group. In **a**, **c** statistical analysis was performed by Mann–Whitney U test. In **b** statistical analysis was performed by non-paired t test

As shown in Fig. 6, latency of passive avoidance test, CBF, and hippocampal CA1 neuronal cell number in wild-type mice were not significantly affected by linagliptin treatment.

**Fig. 7:**
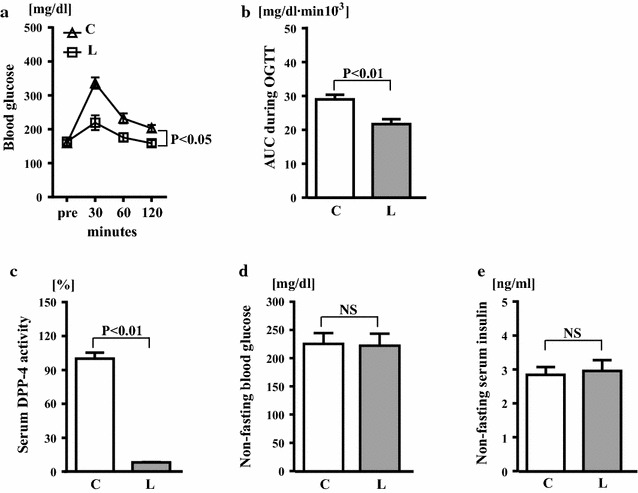
Effects of linagliptin treatment on glucose tolerance (**a**), area under curve (AUC) during oral glucose tolerance test (**b**), serum DPP-4 activity (**c**), non-fasting blood glucose (**d**), and non-fasting serum insulin (**e**) of wild-type mice. Abbreviations used are the same as in Fig. 2. Values are mean ± SEM. In **a**–**c**, **e** n = 7 in each group. In **d** n = 10 in each group. In **a** statistical analysis was performed by one-way repeated measurement ANOVA with Bonferroni correction. In **b**, **d**, **e** statistical analysis was performed by non-paired t test. In **c** statistical analysis was performed by Mann–Whitney U test

Blood glucose levels and AUC during OGTT (Fig. 7a, b) in wild-type mice were significantly lower in linagliptin group than in control group (P < 0.05 and P < 0.01, respectively). Linagliptin treatment almost completely inhibited serum DPP-4 activity in wild-type mice but did not affect non-fasting blood glucose or serum insulin levels (Fig. 7c–e).

## Discussion

Based on multiple lines of experimental evidence [1–6, 27], DPP-4 inhibition is supposed to have multiple protective effects against cardiovascular and renal injuries independently of blood glucose-lowering effect. For example, linagliptin prevents western diet-induced vascular abnormalities in female mice [28], and even reverses western diet-induced cardiac diastolic dysfunction, possibly by targeting TRAF3IP2 expression and its downstream inflammatory signaling [29]. Although diabetes significantly accelerates human premature aging such as brain aging or vascular aging, it is unknown whether DPP-4 inhibition can slow the progression of human premature aging. Therefore, in the present work, we for the first time examined the effect of DPP-4 inhibition with linagliptin on premature aging in klotho−/− mice, one of the most popular animal models of premature aging. The major findings of our present study were that linagliptin significantly ameliorated the progression of premature aging phenotypes, as shown by the amelioration of cognitive impairment, alopecia, body weight gain–loss, and hypoglycemia. Furthermore, there was a trend for linagliptin to prolong survival of klotho−/− mice. These effects of linagliptin in klotho−/− mice were not observed in control wild-type mice, thereby showing that these beneficial effects of linagliptin were specific for klotho−/− mice. Collectively, our present work provided the first evidence suggesting that DPP-4 inhibition might protect against premature aging independently of blood glucose-lowering effect, and provided a novel insight into the potential role of DPP-4 in the underlying mechanisms of premature aging. However, further study is needed to elucidate whether the present findings on linagliptin can be the class effects of DPP-inhibitors or not.

### Amelioration of brain aging by linagliptin and its potential mechanisms

It is established that klotho−/− mice display brain aging characterized by cognitive impairment and hippocampal neurodegeneration [13, 16, 30, 31], although klotho−/− mice have no Alzheimer’s disease-like pathology such as cerebral amyloid plaque and neurofibrillary tangle. In the present study, we obtained the evidence that linagliptin treatment significantly prevented cognitive impairment in klotho−/− mice, as shown by passive avoidance test. Of note, this amelioration of cognitive impairment by linagliptin in klotho−/− mice was associated with the prevention of hippocampal neuronal cell loss and the increase in CBF. The increased CBF in klotho−/− mice by linagliptin seems to be at least in part mediated by the increase in cerebral phospho-eNOS. Therefore, our present work provided the evidence suggesting that DPP-4 inhibition might exert protective effect against brain aging of klotho−/− mice through the suppression of hippocampal neurodegeneration, the improvement of CBF, and the upregulation of brain eNOS. Akt and its downstream molecule, CREB, are well known to be one of the major neuroprotective signaling pathways, and promote hippocampal neuronal cell survival [7, 32–34]. To address the potential role of Akt and CREB in the brain protective effects of linagliptin in klotho−/− mice, we examined the effect of linagliptin on cerebral Akt and CREB of klotho−/− mice. Interestingly, linagliptin treatment significantly increased cerebral Akt and CREB phosphorylation in klotho−/− mice. Collectively, these results suggest that the inhibition of cognitive impairment with linagliptin in klotho−/− mice might be attributed to the activation of cerebral eNOS, Akt, and CREB by linagliptin. Further study is required to elucidate the precise mechanism underlying the amelioration of cognitive impairment by linagliptin in klotho−/− mice.

### Amelioration by linagliptin of not only brain aging but also other various aging phenotypes of klotho−/− mice

Klotho−/− mice are almost indistinguishable from wild-type mice regarding the appearance and the growth up to 3 weeks of age, thereafter they stop growing and body weight gain, develop aging phenotypes, and most of the mice die prematurely at 8–10 weeks of age [12, 15, 35]. However, the mechanism underlying body weight loss in klotho−/− mice is obscure. Furthermore, the specific cause of premature death in klotho−/− mice is unclear since each of these aging phenotypes appears not fatal by itself. In the present study, DPP-4 inhibition with linagliptin significantly slowed body weight loss in klotho−/− mice, and greater body weight of klotho−/− mice treated with linagliptin was associated with greater skeletal muscle weight and greater kidney weight and with the prevention of hair loss. Furthermore, linagliptin treatment tended to prolong survival rate of klotho−/− mice. All these findings suggest that DPP-4 inhibition might exert multiple antiaging effects in klotho−/− mice. However, further detailed investigation is required to define the exact role of DPP-4 in the mechanism of premature aging.

Hypoglycemia is also another aging-related phenotype observed in klotho−/− mice [14]. In the present study, compared with wild-type mice, blood glucose levels of klotho−/− mice were very low and maintained at low levels throughout the OGTT, and this finding is in good agreement with the previous report [14]. Of note are the observations that linagliptin significantly improved hypoglycemia in klotho−/− mice, as shown by the findings of OGTT and significantly increased non-fasting blood glucose levels, while linagliptin did not affect serum insulin levels of klotho−/− mice. Thus, linagliptin treatment significantly ameliorated hypoglycemia in klotho−/− mice, probably independently of insulin. However, the present study did not allow us to elucidate the underlying mechanism of amelioration of hypoglycemia by linagliptin in klotho−/− mice. Furthermore, metabolic cage study could not be technically performed in the present study, since individual housing of klotho−/− mice shortens life span because of much vulnerability to stress.

### Clinical implication

It has been shown that DPP-4 inhibitor-treated type 2 diabetic patients had lower risks for cardiovascular disease as compared to those for non-DPP-4 inhibitor users, except metformin users [36], and its use did not increase the risk of heart failure compared with sulfonylurea [37]. The initial combination of linagliptin and metformin substantially improved glycemic control without weight gain and with infrequent hypoglycemia [38] and also significantly improved microvascular function in the fasting state [39]. Very importantly, type 2 diabetes is known to significantly accelerate premature aging of systemic organs. Taken together with our present findings on benefit of linagliptin in premature aging mouse, all these findings support the notion that DPP-4 inhibitors may be a promising anti-diabetic agent for prevention of cardiovascular disease in type 2 diabetic elderly patients. In conclusion, we for the first time examined the impact of DPP-4 inhibition with linagliptin on premature aging by using klotho−/− mice, a popular animal model of human premature aging. We found that DPP-4 inhibition with linagliptin ameliorated cognitive impairment and hippocampal neurodegeneration, improved CBF, prevented hair loss, lessened body weight loss, and ameliorated hypoglycemia in klotho−/− mice. Thus, our present work provided the evidence suggesting that DPP-4 inhibition might exert anti-aging effects on multiple organs and provided a novel insight into the potential role of DPP-4 inhibition in premature aging.

## Abbreviations

CBF: cerebral blood flow
CREB: cAMP response element binding protein
DPP-4: dipeptidylpeptidase-4
GLP-1: glucagon-like peptide-1
OGTT: oral glucose tolerance test
RPM: rotation per minute
